# Histone Deacetylase Inhibitors: A Promising Therapeutic Alternative for Endometrial Carcinoma

**DOI:** 10.1155/2021/7850688

**Published:** 2021-11-12

**Authors:** Iason Psilopatis, Alexandros Pergaris, Constantinos Giaginis, Stamatios Theocharis

**Affiliations:** ^1^First Department of Pathology, Medical School, National and Kapodistrian University of Athens, 75 Mikras Asias Street, Bld 10, Goudi, 11527 Athens, Greece; ^2^Charité-University School of Medicine, Augustenburger Pl. 1, 13353 Berlin, Germany; ^3^Department of Food Science and Nutrition, University of Aegean, Lemnos, Greece

## Abstract

Endometrial carcinoma is the most common malignant tumor of the female genital tract in the United States. Epigenetic alterations are implicated in endometrial cancer development and progression. Histone deacetylase inhibitors are a novel class of anticancer drugs that increase the level of histone acetylation in many cell types, thereby inducing cell cycle arrest, differentiation, and apoptotic cell death. This review is aimed at determining the role of histone acetylation and examining the therapeutic potential of histone deacetylase inhibitors in endometrial cancer. In order to identify relevant studies, a literature review was conducted using the MEDLINE and LIVIVO databases. The search terms *histone deacetylase*, *histone deacetylase inhibitor*, and *endometrial cancer* were employed, and we were able to identify fifty-two studies focused on endometrial carcinoma and published between 2001 and 2021. Deregulation of histone acetylation is involved in the tumorigenesis of both endometrial carcinoma histological types and accounts for high-grade, aggressive carcinomas with worse prognosis and decreased overall survival. Histone deacetylase inhibitors inhibit tumor growth, enhance the transcription of silenced physiologic genes, and induce cell cycle arrest and apoptosis in endometrial carcinoma cells both in vitro and in vivo. The combination of histone deacetylase inhibitors with traditional chemotherapeutic agents shows synergistic cytotoxic effects in endometrial carcinoma cells. Histone acetylation plays an important role in endometrial carcinoma development and progression. Histone deacetylase inhibitors show potent antitumor effects in various endometrial cancer cell lines as well as tumor xenograft models. Additional clinical trials are however needed to verify the clinical utility and safety of these promising therapeutic agents in the treatment of patients with endometrial cancer.

## 1. Introduction

The nucleosome is the building block of DNA structural organization and enables the necessary packaging of the genetic material in a denser form fitting within the eukaryotic nucleus. It refers to a negatively charged DNA strand wrapped around a positively charged histone octamer, a protein core consisting of two identical copies of each of the four core histone proteins (H2A, H2B, H3, and H4) [1, 2]. In this condensed formation, histones have low levels of acetylation on the lysine residues of their aminoterminal tails, thus blocking the assembly of the basal transcriptional factors to form the preinitiation complex that allows genetic expression [3, 4]. The post-translational modification of the NH2-terminal tails of histones by acetylation neutralizes the positive charge on lysine residues and reduces the affinity of histone for the negatively charged DNA. As such, DNA strands may uncoil and transcription may occur [5]. The level of histone acetylation is modulated by the opposing actions of histone acetylases (HATs) and histone deacetylases (HDACs) [6]. HDACs catalyze the removal of acetyl groups on the NH2-terminal lysine residues of core nucleosomal histones, which generally results in transcriptional repression and silencing of tumor-suppressor genes [7, 8]. Consequently, deregulation of histone acetylation can promote the development of certain human cancers, as shown by a great number of researchers who focused on revealing the link between histone acetylation/deacetylation and carcinogenesis [9, 10].

Endometrial carcinoma (EC) is the most common malignant tumor of the female genital tract in the United States. According to the American Cancer Society, about 66,570 new cases of cancer of the body of the uterus will be diagnosed and about 12,940 women will die from cancers of the uterine body in the United States in 2021 [11]. EC primarily affects postmenopausal women aged 55-64, with the median age at diagnosis being 63 years [12]. ECs can be divided into two distinct histopathologic subgroups: type I EC deriving from atypical endometrial hyperplasia and type II EC of non-endometrioid histology [13]. Type I EC is directly related to long-term exposure to increased estrogen levels and is associated with *PTEN* inactivation by mutation, microsatellite instability, and mutations of *K-ras*, *β-catenin*, or *hMLH1/MSH2*. Type II EC is mostly estrogen-independent, develops from atrophic endometrium in postmenopausal women, and is characterized by *p53* mutations, display inactivation of p16 and E-cadherin, as well as Her2/neu amplification [14, 15]. While surgery is recommended as a monotherapy for low-risk ECs, adjuvant chemotherapy should be offered to women with high-intermediate- and high-risk ECs, as well as advanced or recurrent disease [16]. Combined chemotherapy with carboplatin and paclitaxel is the first-line regimen, followed by chemotherapeutic agents such as doxorubicin, cyclophosphamide, or cisplatin [17].

Despite the reported high response rates, the duration of response is only short-lasting, ranging from between four and eight months [18] and 5-year overall survival amounting to 81%, according to the American Cancer Society [19]. However, prognosis for patients with advanced disease remains grim, with 5-year survival rates dropping to 17% when distant metastasis is present [19]. Such statistics render imperative the development of innovative agents for the effective treatment of EC.

**Figure 1:**
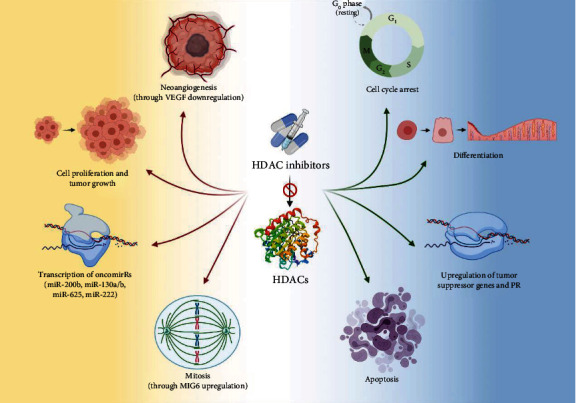
HDAC inhibitors exert their tumor-suppressive role through various mechanisms. Green arrows: procedures enhanced by HDAC inhibitors. Red arrows: procedures blocked by HDAC inhibitors (created with http://Biorender.com). HDAC: histone deacetylase; PR: progesterone receptor; MIG6: mitogen-inducible gene 6; VEGF: vascular endothelial growth factor.

Histone deacetylase inhibitors (HDACIs) are a novel class of anticancer drugs that increase the level of histone acetylation in many cell types, thereby inducing cell cycle arrest, differentiation, and apoptotic cell death, thus suppressing carcinogenesis [20, 21] (Figure 1). Few HDACIs have already received FDA approval for T-cell lymphoma or multiple myeloma, yet there is a great number of current clinical trials investigating the role of HDACIs (alone or in combination with other anticancer drugs) in the treatment of numerous solid cancer entities [22, 23]. Given the genetic alternations in EC, HDACIs could be considered promising therapeutic agents.

### 1.1. Histone-Mediated Epigenetics in EC Clinical Samples and Cell Lines

Histone-mediated epigenetics plays an established role in EC development and progression. A large number of studies have assessed the genetic alternations associated with histone-mediated epigenetics in population-based cohorts of EC tumor types [15, 24–31] (Table 1).

Histone acetylation is involved in the silencing of human mutL homolog 1 (hMLH1)/mutS homolog 2 (MSH2), phosphatase and tensin homolog (PTEN), and progesterone receptor (PR), thus resulting in early carcinogenesis, more aggressive carcinomas, and resistance to hormonal treatment, respectively [15]. Specifically, silencing of hMLH1 and/or MSH2 causes microsatellite instability, invasive growth, and acquired resistance to cisplatin in EC [24]. Class I HDACs (HDAC1, HDAC2, and HDAC3) are expressed in the majority of ECs at high levels, with high-grade serous subtypes exhibiting overexpression of all three HDACs significantly more often than endometrioid subtypes [25]. Notably, HDAC2 overexpression has been suggested to be involved in the acquisition of aggressive behavior by EC [26]. Krusche et al. reported that, compared to normal endometrium, many ECs showed impaired HDAC1 protein expression in the epithelial and stromal compartment, which might be indicative of an impaired epigenetic status of epithelial and stromal cells within ECs [27]. HDAC6, modulated by miR-206, promotes EC progression through the PTEN/AKT/mTOR pathway [28]. Deregulating E-cadherin correlates with focal adhesion kinase (FAK) signaling axis and HDAC/enhancer of zeste homolog 2 (EZH2) activity. EZH2, FAK, and phospho-FAK (pFAK) overexpression is mainly identified in type II ECs and is associated with worse prognosis and decreased overall survival [29]. Low forkhead box A1 (FOXA1) protein expression significantly correlates with high-grade carcinoma, loss of estrogen receptor *α* (ER*α*) and PR, and poor survival [30]. The bromodomain-containing gene ATPase family AAA domain containing 2 (ATAD2) is a mediator of MYC transcriptional function and represents a marker of aggressive ECs [31].

Several in vitro studies have examined the role of histone-mediated epigenetics in EC cell lines as well.

Mitogen-inducible gene 6 (MIG6) mRNA levels are lower in cell lines derived from high-grade ECs than in low-grade EC cell lines. MIG6 is an essential downstream component of PR-mediated growth suppression [32]. Aberrant expression of miRNAs including miR-200b, miR130a/b, miR-625, and miR-222 is associated with tumorigenesis and metastasis in EC cell lines [33].

All of the aforementioned genetic alternations in ECs are strongly influenced by histone-mediated epigenetics.

### 1.2. In Vitro Effects of HDACIs on EC Cell Lines

**Figure 2:**
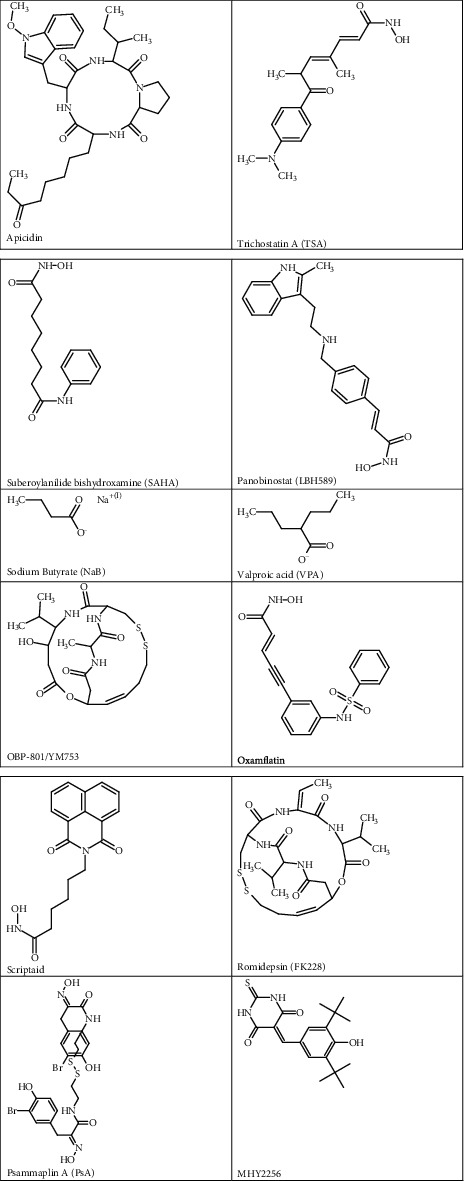
Chemical structures of HDACIs used in EC treatment studies.

There are five identified classes of HDACIs including organic hydroxamic acids, short-chain fatty acids, benzamides, cyclic tetrapeptides, and sulfonamide anilides [14, 34]. Different in vitro studies have investigated the effects of various HDACIs on genetic alternations in EC cell lines associated with histone-mediated epigenetics (Table 2). The reported HDACIs seem to have a profound effect on cell viability by inhibiting cell proliferation and inducing cell death in EC. The specific chemical structures of HDACIs used in EC-related studies are depicted in Figure 2.

*Apicidin*. Apicidin is a fungal metabolite shown to exert antiparasitic activity by the inhibition of HDAC [35]. In EC cell lines, Apicidin results in the upregulation of acetylated H3 and H4, p21, p27, and E-cadherin and the downregulation of cyclin A, cyclin D1, cyclin E, CDK2, CDK4, p53, HDAC3, and HDAC4. As a result, Apicidin induces morphological changes, increases the proportion of cells in the G1 phase, and decreases the number of cells in the S phase [18, 36, 37]. Moreover, Apicidin increases the level of PARP cleavage and caspase-3 activity, induces cytoplasmic localization of cytochrome c, and causes the downregulation of the antiapoptotic gene, Bcl-2, and upregulation of the proapoptotic gene, Bax, thus inducing apoptotic cell death [18, 36]. Concerning estrogen-dependent cancers, Apicidin suppresses transcription of 17*β*-hydroxy steroid dehydrogenase type 1 in EC cells, which is responsible for intratumoral estrone to 17*β* estradiol conversion [38].

*Trichostatin A (TSA)*. TSA, an antifungal antibiotic initially isolated from Streptomyces hygroscopicus, is a potent and specific HDACI [39]. TSA increases the levels of acetyl H3, acetyl H4, acetyl tubulin, p21, p27, miR-130b, DICER1, BIM, L1CAM, FOXA1, glycodelin, and E-cadherin and decreases the levels of cyclin A, cyclin D1 and D2, MMP2, MMP9, DNMT3B mRNA, ER*α*, and MCM7 mRNA [26, 30, 33, 40–46]. After treatment with TSA, cleavage of PARP and caspase-3 was observed, indicating its apoptotic effects [26, 46]. TSA inhibits cell proliferation by arrest in the G1 and/or G2 phases of the cell cycle [33, 46]. Raeder et al. suggested that dependency on MYC predicts dependency on ATAD2 and response to TSA in EC [31], while Zhao et al. demonstrated that the downregulation of MYC in the presence of TSA resulted in the reduction of miR-106b-93-25 cluster [46]. TSA acts synergistically with aza-deoxycytidine and results in a robust and sustainable PR-B upregulation [47]. High-glucose condition and TSA induce degradation of CLDN-2 in Sawano cells [48]. TSA in combination with paclitaxel induces synergistic cell death, results in significant morphologic changes, induces activation of the intrinsic mitochondria-dependent apoptotic pathway, and stabilizes microtubules [49, 50].

*Suberoylanilide bis hydroxamine (SAHA, Vorinostat)*. Vorinostat is a HDACI that reacts with and blocks the catalytic site of HDACs [51, 52]. SAHA induces the activation of caspase-8 and caspase-9, results in the upregulation of glycodelin and acetylated H3 and H4 bound to either Tig1 or C/ebpa gene, downregulates the expression of Bcl-2, cyclin D1, and D2, increases the levels of FOXA1, E-cadherin, p21, and p27, causes a dramatic decrease of FLIP mRNA and protein levels, and induces apoptosis in EC [30, 40, 41, 53–56]. Sarfstein et al. examined SAHA's mechanism of action in type I and type II EC cell lines in the presence or absence of IGF-I and found out that Vorinostat exhibits a potent apoptotic and antiproliferative effect in both type I and II EC cells through interaction with the insulin-like growth factor signaling pathway [57]. SAHA is also effective at reducing AURKA expression in EC, a cell-cycle-regulated kinase that functions in spindle formation and chromosome segregation during the M phase of the cell cycle [58].

*Panobinostat (LBH589)*. LBH589 is a potent pan-deacetylase inhibitor [59]. Treatment with LBH589 induces a profound upregulation of PR mRNA and MIG6, cell cycle arrest in G1, and a downregulation of the oncogene *MYC* [32, 60, 61]. Knockdown of metadherin sensitizes EC cells to cell death induction by death receptor ligand TRAIL and LBH589 co-treatment [62] while the combination of proteasome and LBH589 overcomes the impact of gain-of-function p53 mutations [63].

*Sodium butyrate (NaB)*. NaB is a part of the metabolic fatty acid fuel cycle that also acts as a HDACI [64]. NaB induces upregulation of p21, p27, acetyl H3, and H4 and inhibition of transcription from multiple ER*α* promoters, cell cycle arrest, and apoptosis [41, 42, 65]. The addition of NaB significantly enhances adriamycin cytotoxicity for the primary EC cells with high human telomerase reverse transcriptase expression [66]. NaB has been also reported to inhibit the self-renewal capacity of endometrial tumor side-population cells by promoting the production of intracellular ROS and by upregulating the expression of the phospho-p38 mitogen-activated protein kinase, *γ*H2AX, acetyl H3, p21, and p27 [67, 68].

*Valproic acid (VPA)*. VPA is a HDACI approved for the treatment of epilepsy [24, 69]. VPA inhibits proliferation, induces cell cycle arrest, enhances the apoptotic index in EC cell lines, upregulates E-cadherin mRNA and protein levels, and downregulates Bcl-2 mRNA levels [56]. Moreover, VPA enhances the action of antiestrogens in ER*α*-positive breast cancer cells and blocks tamoxifen-induced proliferation of uterine cells [70]. Cotreatment with VPA and the Aurora kinase inhibitor VE465 induces enhanced apoptosis, cleaved PARP, and cytotoxic effects in EC cells [71].

*OBP-801/YM753*. Combination of the novel HDAC inhibitor OBP-801/YM753 and the PI3K inhibitor LY294002 synergistically induces apoptosis in human EC cells due to increase of BIM with accumulation of ROS [72].

*Oxamflatin*. Oxamflatinis is a HDACI that induces transcriptional activation of jun D and morphological reversion in v-Kras-transformed NIH3T3 cells [73]. Administration of Oxamflatin causes morphologic changes, loss of mitochondrial membrane potentials, and cleavage of PARP, caspase-8, and caspase-9, confirming the activation of apoptotic cascades in EC cells [15].

*Scriptaid*. Scriptaid is a potent HDACI with a >100-fold increase in histone acetylation, with relatively low toxicity [74]. Exposure to Scriptaid decreases the proportion of cells in the S phase, increases the proportion in the G0/G1 and/or G2/M phases of the cell cycle, upregulates the expression of E-cadherin, acetyl-H3 and acetyl-H4, p21, and p27, downregulates the expression of cyclin A and Bcl-2, and induces apoptosis in EC cells [75].

*Romidepsin (FK228)*. FK228 is a HDACI which has been confirmed as a useful anticancer agent [76]. In EC cell lines, FK228 induces apoptosis and cell cycle arrest at G0/G1 phase, increases the mRNA and protein expressions of p53, p21, cleaved caspases such as 3, 7, and 8, and PARP, and upregulates the acetylation of H3 and H4 [77].

*Psammaplin A (PsA)*. PsA is a natural bromotyrosine derivative from a two-sponge association, Poecillastra sp. and Jaspis sp., which was first isolated from the Psammaplysilla sponge. PsA induces the expression of acetylated H3 and H4 histone proteins, upregulates the expression of cyclin-dependent kinase inhibitors and p21, and downregulates the expression of p53, pRb, cyclins, and CDKs, which lead to induce cell cycle arrest [78].

*MHY2256*. MHY2256 is a novel HDACI that inhibits class III HDAC sirtuin (SIRT). MHY2256 reduces both SIRT1 enzyme activity and SIRT protein levels in EC cells, inhibits cell cycle distribution, increases p53 levels, reduces the expression of mouse double minute 2 (MDM2), and induces apoptotic/autophagic cell death [79].

Takai et al. have summarized the half maximal inhibitory concentrations (IC50) of the different classes of HDACIs which indicate how much of each HDACI is needed to inhibit in vitro cell growth in EC cell lines by 50% [14].

### 1.3. In Vivo Impact of HDACI Use in EC

Several studies have examined the anti-tumor effect of HDACIs on human EC cells in mouse models (Table 3).

Apicidin downregulates HDAC3 and HDAC4 and suppresses the tumor growth of transplanted Ishikawa cells, the expression of proliferative cell nuclear antigen (PCNA), and vascular endothelial growth factor (VEGF) in tumor xenograft model, respectively [37].

Co-treatment with TSA and paclitaxel results in a significant reduction in tumor weight, increases microtubule stabilization, and induces apoptosis as well as tubulin acetylation in mouse xenograft models [50].

Combination of Vorinostat and caspase-8 inhibition causes a nearly complete inhibition of tumor xenograft growth [53].

NaB results in marked suppression of tumor growth and SA-*β*-gal activity in tumor xenograft models [65].

VPA and MHY2256 significantly inhibit human uterine tumor growth without toxic side effects in mouse models [41, 79]. Notably, VPA inhibits tumor growth, upregulates CDH1 mRNA, and downregulates Bcl-2 mRNA levels *in vivo* [56]. Yoshioka et al. showed that combined treatment with OBP-801/YM753 and LY294002 significantly suppressed tumor growth compared to the control *in vivo* [72].

In a surgical window trial of women with newly diagnosed endometrioid EC, co-treatment with medroxyprogesterone acetate and the HDACI Entinostat resulted in the reduction of PR H-scores and Ki-67 levels [80].

## 2. Conclusions

The present review summarizes the important role of HDACs in EC development and progression and highlights the potent antitumor effects of various HDACIs on EC cell lines both *in vitro* and *in vivo*. HDACs seem to be involved in the tumorigenesis of both EC tumor types and account for high-grade, aggressive carcinomas with worse prognosis and decreased overall survival. HDACIs represent promising therapeutic agents that inhibit tumor growth, enhance the transcription of silenced physiologic genes, and induce cell cycle arrest and apoptosis in EC cells. Notably, the combination of HDACIs with traditional chemotherapeutic agents shows synergistic cytotoxic effects in EC cells. Nevertheless, clinical trials are needed to verify the clinical utility and safety of HDACIs in the treatment of women with EC, to investigate possible adverse side effects following their administration to patients and to assure their effectiveness depending on HDAC expression by EC cells.

## Figures and Tables

**Table 1:** Genetic alternations in ECs associated with histone-mediated epigenetics.

Genetic alternations in ECs	Impact on EC development and progression	Reference
Silencing of hMLH1/MSH2, PTEN, and PR	Early carcinogenesis, more aggressive carcinomas, resistance to hormonal treatment	[15]
Silencing of hMLH1 and/or MSH2	Microsatellite instability, invasive growth, acquired resistance to cisplatin	[24]
Overexpression of class I HDACs	Significantly more often in high-grade serous subtypes	[25]
Overexpression of HDAC2	Acquisition of aggressive behavior	[26]
Impaired HDAC1 protein expression	Impaired epigenetic status of epithelial and stromal cells	[27]
miR-206 modulation of HDAC6	Progression through the PTEN/AKT/mTOR pathway	[28]
Overexpression of EZH2, FAK, and pFAK	Worse prognosis, decreased overall survival	[29]
Low FOXA1 protein expression	High-grade carcinomas, loss of ER*α* and PR, poor survival	[30]
ATAD2 expression	Aggressive carcinomas	[31]
Low MIG6 mRNA levels	High-grade carcinomas, failure of PR-mediated growth suppression	[32]
Aberrant expression of miRNAs	Tumorigenesis, metastasis	[33]

**Table 2:** In vitro effects of HDACIs on EC cell lines.

HDACI	Upregulatory effects	Downregulatory effects	Synergetic effects	References
Apicidin	Acetylated H3 and H4, p21, p27, E-cadherin, PARP, caspase-3, cytochrome c, Bax	Cyclin A, cyclin D1, cyclin E, CDK2, CDK4, p53, HDAC3, HDAC4, Bcl-2, 17*β*-hydroxysteroid-dehydrogenase type 1	n/a	[18, 36–38]
TSA	Acetylated H3, H4, and tubulin, p21, p27, miR-130b, DICER1, BIM, L1CAM, FOXA1, glycodelin, E-cadherin, PARP, caspase-3	Cyclin A, cyclin D1 and D2, MMP2, MMP9, DNMT3B mRNA, ER*α*, MCM7 mRNA, MYC, miR-106b-93-25	*Aza-deoxycytidine*: PR-B upregulation *High-glucose condition*: degradation of CLDN-2 *Paclitaxel*: cell death induction	[26, 30, 31, 33, 40–50]
SAHA	Acetylated H3 and H4 bound to either Tig1 or C/ebpa gene, caspase-8 and caspase-9, glycodelin, FOXA1, E-cadherin, p21, p27, insulin-like growth factor-I receptor	Cyclin D1 and D2, Bcl-2, FLIP mRNA and protein levels, AURKA	n/a	[30, 40, 41, 53–58]
LBH589	PR mRNA, MIG6	MYC	*Death receptor ligand TRAIL*: cell death induction after knockdown of metadherin *Proteasome*: overcomes the impact of gain-of-function p53 mutations	[32, 60–63]
NaB	Acetylated H3 and H4, p21, p27, ROS, phospho-p38 mitogen-activated protein kinase, *γ*H2AX	ER*α*	*Adriamycin*: high human telomerase reverse transcriptase expression	[41, 42, 65–68]
VPA	E-cadherin	Bcl-2	*VE465*: PARP cleavage induction	[56, 70, 71]
OBP-801/YM753	n/a	n/a	*LY294002*: BIM increase with accumulation of ROS	[72]
Oxamflatin	PARP, caspase-8 and caspase-9	n/a	n/a	[15]
Scriptaid	Acetylated H3 and H4, p21, p27, E-cadherin	Cyclin A, Bcl-2	n/a	[75]
FK228	Acetylated H3 and H4, p21, p53, caspase-3, caspase-7, and caspase-8, PARP	n/a	n/a	[77]
PsA	Acetylated H3 and H4, p21	p53, pRb, cyclins, CDKs	n/a	[78]
MHY2256	p53	SIRT1 enzyme activity, SIRT protein levels, MDM2	n/a	[79]

**Table 3:** Antitumor effects of HDACIs on human EC cells in mouse models.

HDACI	Upregulatory effects	Downregulatory effects	Synergetic effects	References
Apicidin	n/a	HDAC3, HDAC4, PCNA, VEGF, Tumor growth	n/a	[37]
TSA	n/a	n/a	*Paclitaxel*: reduction in tumor weight, increase in microtubule stabilization, apoptosis induction, tubulin acetylation induction	[50]
SAHA	n/a	n/a	*Caspase-8 inhibition*: tumor growth inhibition	[53]
NaB	n/a	SA-*β*-gal activity, Tumor growth	n/a	[65]
VPA	CDH1 mRNA	Bcl-2, Tumor growth	n/a	[41, 56, 79]
MHY2256	n/a	Tumor growth	n/a	[41, 79]
OBP-801/YM753	n/a	n/a	*LY294002*: tumor growth inhibition	[72]
